# Lab-on-a-Chip Platforms as Tools for Drug Screening in Neuropathologies Associated with Blood–Brain Barrier Alterations

**DOI:** 10.3390/biom11060916

**Published:** 2021-06-21

**Authors:** Cristina Elena Staicu, Florin Jipa, Emanuel Axente, Mihai Radu, Beatrice Mihaela Radu, Felix Sima

**Affiliations:** 1 Department of Anatomy, Animal Physiology and Biophysics, Faculty of Biology, University of Bucharest, 050095 Bucharest, Romania; elena.necsulescu@drd.unibuc.ro; 2 Center for Advanced Laser Technologies, National Institute for Laser, Plasma and Radiation Physics, 077125 Măgurele, Romania; florin.jipa@inflpr.ro (F.J.); emanuel.axente@inflpr.ro (E.A.); felix.sima@inflpr.ro (F.S.); 3 Department of Life and Environmental Physics, ‘Horia Hulubei’ National Institute for Physics and Nuclear Engineering, 077125 Măgurele, Romania; mradu@nipne.ro

**Keywords:** lab-on-a-chip, organ-on-a-chip, microfluidic platforms, blood brain barrier, neurovascular unit, drug screening, neurodegenerative disorders

## Abstract

Lab-on-a-chip (LOC) and organ-on-a-chip (OOC) devices are highly versatile platforms that enable miniaturization and advanced controlled laboratory functions (i.e., microfluidics, advanced optical or electrical recordings, high-throughput screening). The manufacturing advancements of LOCs/OOCs for biomedical applications and their current limitations are briefly discussed. Multiple studies have exploited the advantages of mimicking organs or tissues on a chip. Among these, we focused our attention on the brain-on-a-chip, blood–brain barrier (BBB)-on-a-chip, and neurovascular unit (NVU)-on-a-chip applications. Mainly, we review the latest developments of brain-on-a-chip, BBB-on-a-chip, and NVU-on-a-chip devices and their use as testing platforms for high-throughput pharmacological screening. In particular, we analyze the most important contributions of these studies in the field of neurodegenerative diseases and their relevance in translational personalized medicine.

## 1. Introduction

Lab-on-a-chip (LOC) devices are promising microfluidic platforms that allow miniaturization and the integration of multiple laboratory functions. They may accommodate specific components and functions, such as electronics, optics, fluidics, or biosensing structures, at a centimeter/millimeter down to micro- and nanoscale [1,2].

These microdevices are used in different types of laboratory analyses, biochemical operations, DNA sequencing, or chemical synthesis. Among the applications in which LOC platforms can play important roles, one may outline the analysis of ions from different compositions used in fields such as forensics, the identification of explosives, evaluation of water quality, study of body fluids, in agricultural domain or detection of pollution levels [3].

In the last years, the LOC platforms used for biological purposes have been intensely developed, with a special focus on three-dimensional (3D) configurations. While decreasing device sizes, small volumes have significant benefits, which include reduced reagent costs and increased accuracy of analysis. Such biochips, made of glass or polymers, allow biological investigations at the cellular level, including single cell analysis. These 3D in vitro models may represent alternatives for animal sacrifices and in vivo experiments, due to the quasi-realistic reproduction of the physiological systems [4].

Thus, LOCs can be used in different studies targeting organ/tissue models, including the blood–brain barrier, blood vessels, kidney, heart, lung, liver, intestine, muscle, or even tumors [5]. The advantages of this technology rely on increased spatial resolution for interrogation, automated measurements, robustness, low costs, and user-friendly properties [6].

The goal of this review is to summarize the most recent studies on microfluidic platforms mimicking the blood–brain barrier (BBB) and/or the neurovascular unit (NVU), and also to outline the reports focused on the use of these platforms as pharmacological screening tools in neuropathologies.

## 2. LOC Materials and Manufacturing Advancements for Biomedical Research

During the last few decades, microfluidics has triggered various developments in different scientific and technological fields such as disease diagnostics, drugs screening, single-cell analyses, biosensing, analytical chemistry, and micro- and nanofabrication [7,8]. LOC materials are processed by various techniques to develop 3D hollow structures of small dimensions down to the micro- and nanoscale in different complex shapes including channels, chambers, or valves [9]. 

At the same time asthe broad spectrum of applications diversification, strong advances were achieved in the development of appropriate materials and microfabrication technologies. Briefly, there are six main types of materials currently used for the manufacturing of microchips: silicon/glass, poly(dimethylsiloxane) (PDMS), thermoplastics, thermosets, paper, and more recently, hydrogels [10].

Although the fabrication costs could be high (clean room conditions and/or sophisticated processing equipment are needed), inorganic materials may allow accurate processing with high spatial resolution for microfluidic devices. Organic materials are good alternatives, although they involve multiple technological processing steps, including, casting, molding, replication, bonding, and sometimes limiting usage. Lately, paper microfluidics is focusing on a limited number of applications only, while hydrogels are considered as relevant biomimetic materials for microfluidic assays, which are also suitable for 3D bioprinting. The main physical–chemical properties of these materials and current processing technologies employed for device fabrication are summarized in Table 1.

Silicon and glass were the first materials used to develop LOC platforms [13]. The technologies used in the fabrication of microfluidic biochips have expanded during the last few years [14]. Since silicon is expensive and optically opaque in the visible spectrum, there are some limitations for its biological use. Polymers appeared as a relevant alternative and contributed to a rapid advancement of the microfluidics field. Then, the non-photolithographic micro- and nanofabrication of micro-systems was possible in regular laboratory rooms, without the need for clean room equipment. This involved using elastomeric stamps to create patterns with feature sizes down to a few tens of nanometers [15]. Microfluidic systems made of PDMS, an optically transparent soft elastomer, were then the most employed structures with characteristics exploited to control various patterns and microchannels relevant to biology for cellular studies [1]. We further present PDMS and glass-based LOCs as they may offer a good trade-off between flexibility to be processed, transparency, biocompatibility, range of applications, and costs.

### 2.1. PDMS LOCs

PDMS is the most used material in microfluidics for LOC applications due to its relative facile fabrication and relevant properties such as resistance at chemical, physical, or biological agents [16]. The PDMS material confers a number of advantages: it is biocompatible, cheap, easy to model, transparent, and facilitates biological studies on cell cultures due to its properties regarding gas and water permeability [9,17,18,19,20,21,22,23]. 

PDMS surfaces are rather hydrophobic, but they may become hydrophilic by oxygen plasma treatment [24], modification using oxygen and C2F6, using oxygen plasma polymerization of 2-hydroxyethyl methacrylate (HEMA) [25], using atmospheric RF plasma [26], by oxygen plasma treatment, followed by treatment in deionized water [27], corona/air plasma [28], or even surface treatment with NaOH, especially when it comes to microchannels [29]. Indeed, the main drawback of PDMS, in particular for biomedical applications, resides in its hydrophobic properties (poor surface wetting and heterogeneous charge), which may further induce the undesired adsorption of organic molecules. On the other hand, there are several approaches that addressed hydrophilicity conservation by combining UV irradiation and oxygen plasma [30] or chemical grafting treatments [31,32]; that may increase surface wetting stability from tens of minutes up to six months. Simple alternatives for surface hydrophilicity conservation also include the storing of PDMS under water [33] or at very low temperatures [34] after oxygen-based plasma treatment. All these aspects must be carefully addressed in correlation with the required channel geometry for the envisaged application. 

**Figure 1:**
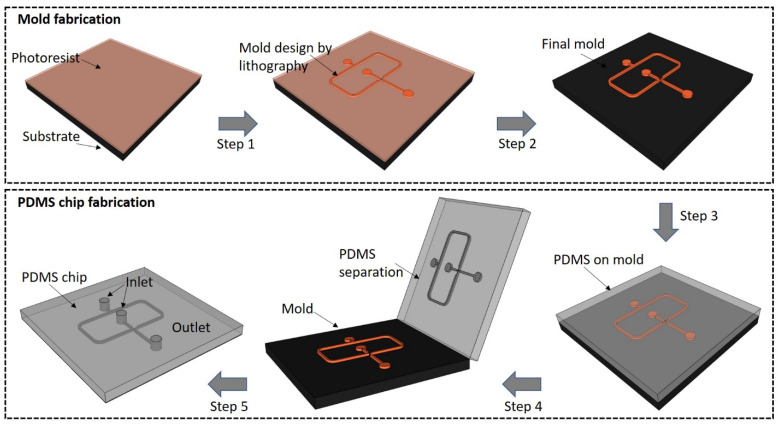
Schematic representation of a droplet generator microchip based on the technology used in our laboratory: ultrashort pulsed laser lithography applied for the mold fabrication (steps 1 and 2) followed by PDMS casting (steps 3–5).

The PDMS LOCs are intensively used in either 2D or 3D configurations [35]. They are relatively easy to be manufactured by lithographic techniques in rather short times and at minimal costs [36,37]. Specifically, the process of a chip production by photolithography consists of a mold fabrication with a desired geometrical configuration chosen for a specific application. The mold can be obtained by the direct light irradiation of a photoresist followed by chemical development of the material to obtain the desired design and subsequent PDMS casting to create the microfluidic chips [36] (schematic example shown in Figure 1).Although the process is rather laborious and time consuming due to several technological steps, the mold could be reused for the replication of several biochips with high accuracy. Two-photon polymerization (TPP) is a laser lithographic technology that employs ultrashort pulsed lasers to fabricate polymeric structures with nanoscale resolution. The polymerization is initiated by a laser beam focused through an objective onto a photoresist material. When applied to negative-tone photoresists, TPP is considered an additive processing technique because the polymerization occurs throughout the scanning trajectory of the focused laser beam while non-exposed areas are washed away by solvents. Theoretically, there is no limitation of resolution due to the material threshold effect correlated with the precision control of the high peak laser intensity, so that sub-100 nm features can be obtained [38]. However, this technique may not be appropriate for large area processing but rather for downsizing dimensions in microfluidic platforms.

A limitation in the manufacture of these microdevices arises when true 3D structures are desired, since more than one mold is needed. Therefore, several attempts have been employed to obtain chips with 3D microenvironments [37]. One approach is to create individual 2D structures that are further interconnected and bonded together using air plasma [39] or oxygen plasma [40,41,42]. Although successful, this method is time consuming and requires high precision in the construction and alignment of the parts, which could be a strong drawback. Another approach is to use the new 3D printing technologies for direct writing of the structures without requiring too much intervention of the users. However, the equipment is still expensive, and the dyes used in the printing may render the polymer opaque, thus limiting the optical interrogation [37]. 

### 2.2. Glass LOCs

**Figure 2:**
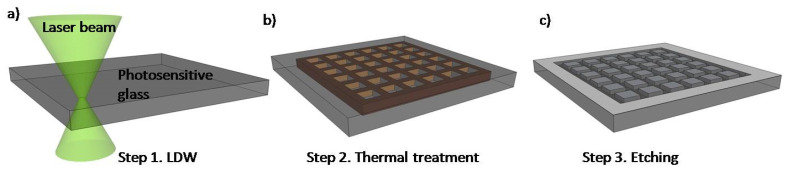
Schematic of photosensitive glass processing based on the technology used in our laboratory: (**a**) picosecond laser direct writing (LDW); (**b**) thermal annealing and (**c**) selective glass etching.

Due to high chemical and temperature resistance, inertness to many substances, and low nonspecific adsorption, glass materials are of great interest for microfluidic applications in biology [43]. Glass exhibits a high degree of transparency and can be rather easy to be processed either by chemical or physical methods.The use of glass in the manufacturing processes of microfluidic devices offers some important advantages over polymeric materials, such as robustness, higher optical quality, or low adsorption of organic compounds. Wet or dry chemical etching techniques or mechanical processes can be applied for the fabrication of glass micro-scale devices but with low precision and productivity as compared to lithographic processes [43]. The use of lasers in combination with a liquid environment allows glass machining with better control over heat and crack. Depending on the final application, glasses such as quartz, borosilicate glass (Pyrex) [44], soda lime glass [45], or photosensitive glass can be used to manufacture both 2D and 3D free-form microfluidic devices, usually through a laser irradiation process, followed by wet or dry etching, in which the exposed region is removed with high selectivity. Glasses can be also bonded with PDMS to form complex 3D structures or microfluidic connections. This bonding is achieved by various methods such as oxygen plasma treatment [46], oxygen plasma followed by heat treatment [47], air plasma/corona [48], or using chemical crosslinking agents [49]. These glass–polymer hybrid structures can be used for specific biology studies, with increased capabilities of reproducing physiological environments [50]. Photosensitive glasses are a category of glasses that allow microfabrication by UV or laser irradiation followed by etching for microfluidic applications. Femtosecond and picosecond laser-assisted etching are subtractive 3D processing methods that use laser direct writing, thermal treatment, and subsequent chemical wet etching to fabricate true 3D hollow channels inside glass (Figure 2) [51,52]. 

Thus, it is then possible to fabricate complex, 3D channels in glass for specific biomicrofluidic applications [53,54]. By laser technologies, one may create microfluidic circuits even on large areas, without supplementary steps of stacking or bonding while specific properties of glass such as robustness, portability, and transparency are preserved. Such glasses are biocompatible, easy to clean, and consequently reusable as 3D biochips or even molding systems [52,55]. A heat treatment can be applied to these materials to obtain a very smooth surface necessary to create relevant cellular environments [56,57].

On the other hand, hybrid subtractive and additive processing can be combined to develop functional polymeric structures inside robust, highly transparent glass microchannels. Specifically, subtractive laser etching of glass followed by the additive polymerization of negative photoresists can be applied to fabricate polymeric 3D microstructures inside embedded glass microfluidic channels [58]. Thus, one may downsize, below 1 μm, dimensions of various 3D complex objects while improving the structure stability [57]. This process allows users to customize complex designs to obtain reliable 3D biochips for concrete applications.

### 2.3. Biomedical Applications of LOCs

It is common knowledge that 2D cell culture and animal models exhibit limited predictability for drug discovery, and therefore, there is an urgent need to find better models for efficient and reproducible drug screening. On the other hand, the ethical rules governing the experiments involving laboratory animals became more and more restrictive, limiting the in vivo preclinical analysis extent. In this context, LOC platforms seem to be a robust technology with extended customization possibilities that can replace the standard cell cultures and animal models in biomedical approaches. Organ on-a-chip (OOC) is a well-established transdisciplinary technology that is facing challenges at aiming to develop microfluidic-based perfusion devices able to mimic the keyfunctions of a specific organ/tissue in both normal and pathological microphysiology [59].

To date, several organs and tissues have been mimicked on a chip (see Table 2), including alveolus [60], bone marrow [61], gut [62], heart [63,64], lung [65], pancreas [66], skin [67], or complex interactions between tissues have been integrated on a chip, such as lung–liver–heart [68] or intestine–liver–brain–kidney [69]. Such OOC applications are targeting the analysis of the cellular behavior in physiological conditions, the development of screening platforms to test the cellular response to various drugs or stimuli, or the in vitro modeling of a pathological condition (e.g., inflammation, edema etc.). 

A comprehensive review devoted to recent advances in the field of organ-on-a-chip engineering was reported by Zhang et al. [35]. The authors discuss how OOC technology can mimic the keyfunctions of organs, in close relation with human physiology, by focusing on tissue barrier properties, parenchymal tissue function, and multi-organ interactions. In a different study, Maschmeyer et al. developed a four-organ-chip for interconnected long-term co-culture of human intestine, liver, skin, and kidney equivalents [71], and they showed the preservation of the microphysiological functionality of the system over 28 days. In a critical review, Junaid et al. advanced an end-user perspective on the latestOOC developments and highlighted how the validated academic proof-of-concept studies could be translated to real-world societal solutions [72]. The challenges for bridging the gap between lab and industry in the field of OOC technologies were recently addressed by Ramadan and Zourob [59]. OOC is a well-recognized multidisciplinary approach that is expected to change many aspects of preclinical-to-clinical translation in the biomedical field. However, there are still many scientific and technical challenges, as well as standardization and regulatory endorsement that should be overcome before technological transfer and commercialization of OOC microdevices.

In the last years, several studies have focused their attention on employing the OOC technology to obtain brain-on-a-chip devices. An integrative review describes the strategies of fabrication for brain-on-a-chip devices and their relevance/compliance as testing platforms for pharmacological screening and disease monitoring [73]. Miccoli et al. emphasized the impact of exploiting OOC platforms, instead of animal models, to perform preclinical pharmacological tests and highlighted the importance of using patient-derived neurons for a strong model reliability [73]. Indeed, the best way to achieve a good 2D or 3D microfluidic brain-on-a-chip model is to combine the use human stem cells (e.g., neural stem cells, induced pluripotent stem cells or embryonic stem cells) with advantages of such a device, including the use of a small amount of fluid, the possibility of creating shear stress conditions, and the low costs of production [74]. Further technological developments employed in brain-on-a-chip devices, such as optogenetics, brain organoids, and 3D bioprinting, are also essential, taking into account the challenge of integrating the complexity of neuronal architectures and connectivity (i.e., 52 regions with distinct cellular organization in human brain) on a chip mimicking brain physiology and pathology [75,76]. 

A collection of studies devoted to brain-on-a-chip models is presented in Table 3. The majority of the brain-on-a-chip models are based on organoids/neurospheroids obtained either from human stem cells or primary rodent neuronal cultures [42,77]. In detail, brain-on-a-chip devices have been used to model neurodevelopmental disorders due to prenatal nicotine exposure [77], neurodegenerative disorders [42,78], neural transplantation therapy in severe degenerative brain diseases [79], amyloid-β induced axonopathy [80], etc. More insights on BBB- and NVU-on-a-chip are presented in the next section of this review.

## 3. BBB andNVU on a Chip

The brain is an organ with an extremely sophisticated structure, which requires a large amount of energy that is mainly supplied by blood with the necessary energy substrates (e.g., glucose and oxygen) [81]. In addition, blood transports multiple substances, among which are also waste products (i.e., neurotoxins), whose access inside brain parenchyma should be prevented. In this context, the brain capillaries’ walls form an interface, called the BBB, with a set of structural and functional features that regulate the transport of substances from the blood to the brain and the other way around [82]. This barrier is largely composed of specialized brain microvascular endothelial cells that separate blood from the interstitial fluids of the brain and, together with the choroid plexus and the arachnoid, help maintain brain homeostasis. The BBB also mediates the passive and active transport of the elements, and it plays an important role as an immunological and metabolic barrier [83].

The BBB is part of the NVU, along with neurons, astrocytes, pericytes, microglia, and the extracellular matrix [84,85,86]. To define, the NVU is considered a set of structures that allow the coordinated response between brain parenchyma and cerebral vascular endothelium to be maintained [87]. Neurons are responsible for using/detecting oxygen and nutrient changes and transforming this information into electrical or chemical signals, which they send to astrocytes either directly or through interneurons creating communication networks [86]. Astrocytes, which are five times more abundant than neurons, are important actors of NVU that regulate cerebral blood flow and brain energy metabolism, or are partners in gliotransmission [87,88,89]. Pericytes also play an important role in the NVU, being in direct contact with the brain endothelial cells, offering them support and actively participating in their development and maturation [86]. Microglia are immunocompetent cells of the NVU, acting as pathological sensors, whose activity is to constantly investigate the intracranial environment, and to remove the damaged cells from dysfunctional synapses or any other debris from brain parenchyma [90].

**Figure 3:**
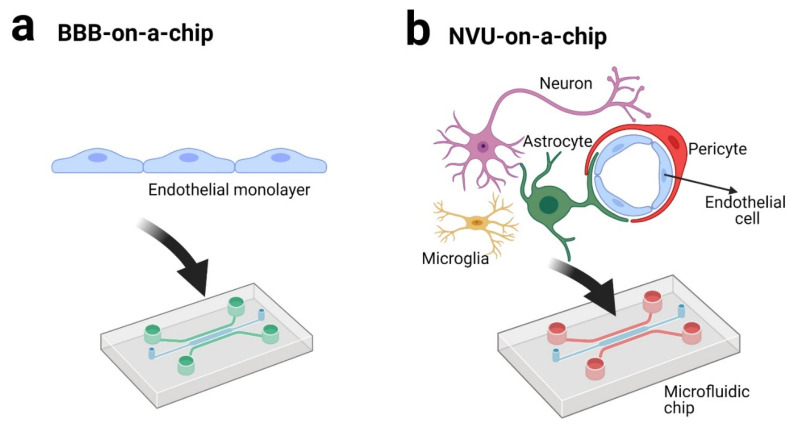
BBB-on-a-chip (**a**) and NVU-on-a-chip (**b**) technologies (created with BioRender.com [91]).

LOC technology has been intensively applied in recent years as BBB-on-a-chip or NVU-on-a-chip technologies (Figure 3).

These technologies have been employed for studying the following (Table 4): the role of BBB in neuroinflammatory, neurodegenerative (e.g., Alzheimer’s, Parkinson’s), or in schizophrenia pathologies [20,22,74,92,93], the interactions between BBB and combinations of cytokines and lipopolysaccharides, leading to loss of function [94,95], the permeability of BBB for drugs or endogenous molecules [17,23,96], the biochemical modulation of BBB [97], the antibody interaction with BBB [23,98], the neuronal–endothelial metabolic coupling [18], or the interaction between cancer cells and astrocytes in a BBB microenvironment [99].

Some of the recent LOC studies devoted to blood–brain barrier summarizing cell sources, functional hallmarks, disease models, and drug tests were reviewed by [35]. Booth and Kim [100], developed a microfluidic blood–brain barrier (µBBB) in order to mimic the dynamic in vivo microenvironment and a comparatively thin culture membrane of 10 µm. The authors proved the validity of the model using co-cultures of bEnd.3 endothelial cells and C8-D1A astrocytes, and they concluded that such system can be used to predict the rate of delivery of new drugs across the BBB, being a valid option for preclinical studies. The research team of Prabhakarpandian et al. demonstrated the similarity between the Synthetic Microvasculature model of BBB (SyM-BBB) and the cerebral microvascularization observed in vivo. They used a microfluidic chip made of PDMS connected to a perfusion system, and as cells, they used rat brain endothelial cell line (RBE4). They performed different tests on cells grown on the surface of the fabricated microdevices; the cultured cells were subjected to astrocyte conditioned media, in the infusion system, for 96 h. Following comparative tests between transwell chambers using porous membranes and SyM-BBB, fluorescence type, Western blot, efflux transporter studies, etc., they concluded that the cells behave on a chip similar to the functional cells in vivo from BBB [112]. Additionally, Jeong et al. tested a 3D arrayed microfluidic BBB-on-a-chip model and integrated an electrical sensor to measure the transendothelial electrical resistance (TEER), and they concluded that their chip mimicked closely the in vivo BBB environment [103]. 

An important aspect that deserves attention is the advantages offered by the BBB-on-a-chip devices in comparison with the traditional transwell system (Table 5). In a study using co-cultures of endothelial cells and astrocytes, Deosarkar et al. demonstrated that the 3D microfluidic platform mimicked the neonatal physiological environment more accurately than the transwell system [113]. Other studies have demonstrated higher resistance values in TEER measurements for the BBB-on-a-chip model compared to the transwell model for brain microvascular endothelial cells with or without astrocytes or pericytes in co-culture [23,95,97,100].These results were similar irrespective of the brain endothelial cells origin (i.e., human or rodent) and demonstrate the superior qualities provided by the BBB-on-a-chip model in comparison to the traditional transwell model in terms of barrier permeability or tight junctions proteins immunostaining.

In comparison to the BBB-on-a-chip model, the NVU-on-a-chip model is more elaborate and requires the use of different types of brain cells (e.g., astrocytes, pericytes, neurons etc.) beside the brain microvascular endothelial cells. Additionally, the calcium signaling machinery of the brain microvascular endothelial cells [114] is strongly influenced by the absence/presence of the NVU adjacent cells. Moreover, the NVU-on-a-chip model implies a complex design with multiple compartments that mimic the blood, brain parenchyma, and cerebral spinal fluid [115]. Several groups have developed NVU-on-a-chip models with innovative architectures. In detail, the different NVU-on-a-chip models were employed to study the neurodevelopment [17,111], the metabolic consequences of inflammatory disruption of the BBB [94], or neurological disorders [110].

An important advantage of the BBB/NVU-on-a-chip is their usefulness in avoiding animal sacrifice for the purpose of these studies. In the future, researchers will try to obtain models on microfluidic chips that are as realistic as possible and perform experiments that mimic the intracranial physiological environment. Although animal models have the great advantage of an intact BBB/NVU with the whole complexity of the brain, there are also major disadvantages including costs, long-term care of animals, and ethical issues [116]. Therefore, despite the obvious limitations, the majority of BBB/NVU functions can be mimicked on a chip, with minimal manufacturing expenses [117], in a variety of configurations and with extended possibilities for drug screening. The progress done so far in using LOC devices as pharmacological screening tools, in particular in neurodegenerative diseases, are detailed in the next sections of this review.

## 4. Microdevices as a Pharmacological Screening Tool

Microfluidic LOCs have the potential to be used as drug testing platforms, to model various diseases, to understand different cellular and molecular mechanisms [9], or for biomarker identification, with the eventual aim of replacing animals in preclinical tests [118]. Thus, LOCs can offer appropriate conditions for the evaluation of cellular activity and drug metabolism, as well as drug efficacy and toxicity [119]. The use of such testing microdevices in pharmacokinetic applications was reported in several studies devoted to the development of new strategies for personalized cancer treatments [12,120,121]. For example, an LOC platform was proposed for the real-time analysis of up to five different drugs simultaneously against osteosarcoma cells [121]. Other LOC microdevices were developed to mimic a hypoxic tumor microenvironment and perform cytotoxic and genotoxic single-cell assays [122]. Anti-inflammatory compounds were also tested in a human lung inflammation ‘small airway-on-a-chip’ model under dynamic flow conditions [123]. It should be emphasized that LOC technology is already used in drug screening and recognized by the United States Food and Drug Administration for testing in the pharmaceutical drug safety industry [124,125]. 

OOC biomimetic systems are proposed to mimic the architectures and functionality of human organs as non-conventional models for testing drug efficacy or safety. In vitro co-culture models may reproduce the complex interactions of cells in an environment similar to that in vivo. By also reproducing microfluidic dynamics, one may exploit these devices in a physiologically relevant manner. In addition to cell co-cultures on the chip, one may take advantage of the sensor integration on the same platform, the controlled infusion of substances, microscopic super-resolution investigation, and high-throughput analysis with reduced time and costs [22].However, a disadvantage of these platforms is that they are customized for specific applications without the possibility of using a platform universally [120]. From the recommendations for standardization, one may consider it critical to focus on mimicking rather single organs and find real benefits and correlate the OOC models with specific local tissue architectures and cellular phenotypes to eventually recapitulate in vivo human physiology [126]. Thus, customizable models for fit-for-purpose OOC with technical and biological modules may be assembled to get standard open technology platforms [127].

The use of the OOC approach to study the efficacy or toxicity of drugs can be carried out on chips made of different materials. To obtain appropriate conditions for several types of tests that indicate the sensitivity of the organs to drugs, the most suitable are biochips of PDMS or glass. These materials, which are rather easy to be processed in complex shapes, offer transparency for optical interrogation and may confer properties similar to the physiological environment, allowing the creation of models of body parts [128]. The usage of these platforms in pharmacological screening studies and drug tests confers the advantage of reducing the number of experiments for several types of organs, such as lungs, liver, central nervous system, kidneys, heart [129], or the study of cancer [128]. LOC microfluidic platforms also have the advantage of being able to test poorly permeable drugs [130].

Indeed, there is still a great need for innovative drug delivery systems, and microfluidics prove to be a cutting-edge technique for this purpose. The advantages of LOCs over conventional methods for the synthesis of advanced delivery systems were reported in several studies. An overview of the droplet microfluidic techniques as a powerful tool for the fabrication of monodisperse drug delivery systems (microcapsules, microspheres, polymersomes, and liposomes) was reported by Fontana et al. [131].

In an Expert Opinion on Drug Safety review paper, Cavero et al. explained the benefits of OOCs microdevices for human-predictive biological insights on drug candidates, with respect to traditional (2D, static) pharmaceutical assays. The authors introduce a broad spectrum of OOC platforms e.g., cancer-, lung-, blood–brain barrier-, heart-, intestine-, kidney-, liver-, pharmacokinetics-, placenta-, and vessel-on-chip, as well as their relevance for drug research and development [132]. The testing strategies may focus on one drug–one or several organs [133] or several drugs–one organ systems [134] and combinations thereof, while some distinct and specific examples are given in the following.

Kim et al. [135] investigated the pharmacokinetic profile that reduces the nephrotoxicity of gentamicin in a kidney-on-a-chip model under dynamic conditions. The goal of the study was to fill the gap between renal clearance in case of humans and animals using a dedicated OOC. It was found that gentamicin alters cell–cell junctions, increases membrane permeability, and decreases cell viability especially during prolonged low-level exposure. 

Three-dimensional (3D) bioprinting was used to fabricate artificial endothelialized myocardium and heart-on-a-chip models for cardiovascular toxicity evaluation [64]. Then, dose-dependent responses of both cardiomyocytes and endothelial cells were evaluated when exposed to doxorubicin. Lind et al. [136] employed multi-material 3D printing to construct cardiac micro-physiological models containing strain sensors within micro-architectures. Isoproterenol and verapamil drugs and the mechanical responses of human stem cell-derived laminar cardiac tissues were examined over four weeks to validate the OOC platform.

A vascularized OOC platform for large-scale drug screening was proposed by Phan et al. [137] to mimic the complexity of in vivo physiology. Thus, several arrays of vascularized micro-tumors were created and tested against up to twelve FDA-approved anti-cancer drugs, revealing the successful identification of both anti-angiogenic and anti-tumor drugs. 

The effect of hepatic metabolism on off-target cardiotoxicity in a multi-organ human-OOC model system was investigated [138]. A co-culture of human primary hepatocytes with iPSc-derived cardiomyocytes was used to test terfenadine and fexofenadine drugs, which are potentially related to cardiac side effects dependent on hepatic metabolism. Theobald et al. reported the development of a liver–kidney-on-chip model to investigate the toxicity of drug metabolites [139]. In vitro drug screening is performed on a platform that allows mimicking the exchange between different organ specific cell types in a flow-dependent manner. The toxicity evaluation of aflatoxin B1 and benzoalphapyrene drugs validated the efficiency of this OOC system. A 3D tetra-culture brain-on-chip platform was proposed for screening organophosphate toxicity [140]. The study revealed the high utility of such platforms by measuring drug effects on barrier integrity.

On the other hand, the emerging role of OOCs in quantitative clinical pharmacology evaluation was reviewed by Isoherranen et al. [141]. It is stated that advances in the micro-physiological system such as OOC technology could bring new insights in predicting drug effects, designing preclinical/clinical trials, and improved personalized treatments.

Very recently, the application of OOC technology for drug discovery was reviewed [142]. When compared with traditional approaches for drug efficacy and testing, the biomimetic OOC system, simulating both the biology and physiology of human organs, has shown greater advantages. It is discussed how a “human-on-chip” system can mimic the complex and dynamic processes such as drug absorption, distribution, metabolism and excretion, and evaluate drug efficacy and toxicity with more reliability.

The merging of microfluidic LOCs with pharmaceutical analysis and pharmacological/toxicological assays was reviewed in detail recently by [143]. The authors summarized the efforts of the scientific community for the development of “Pharm-Lab-on-a-Chip” platforms, which are able to address the whole range of pharmacological advances, from recent drug discovery to post-marketing product management. They focused the literature survey on applications such as the separation and analysis of drugs on a chip, development of new tools for pharmacological/toxicological models on a chip, and the application of chip-based models for screening both the drug’s efficacy and safety [143]. Several perspectives for the future challenges and breakthroughs related to Pharm-Lab-on-a-Chip advances, such as automating drug discovery, precision nanomedicine, and personalized therapy, are then highlighted.

However, a possible bottleneck in the OOCs model implementation in clinical use is related to its still limited resemblance to the real in vivo tissues and a lack in the development of human disease models [144].

## 5. Drug Screening in Neurodegenerative Disorders Using Microdevices

During the last few decades, drug screening became a very powerful tool to select drug candidates for investigated diseases. Placed between the in silico design procedures or drug libraries development and the short list of selected drug candidates, the screening procedures allow the selection of the most promising molecular structures for the targeted pharmacological effects [145]. In particular, chips especially designed to allow mimickingthe NVU structures (some examples are presented in [73]) provide full control of each cell type included in the NVU model. Such approaches have the advantages of assuring access to various molecular targets and controlling the experimental condition in a way that is impossible to attain in in vivo experiments.

Neurodegenerative diseases are commonly associated with the BBB permeabilization. This phenomenon occurs due to the accumulation of proteins on the surface of the endothelial cells. β-amyloid and α-synuclein are two examples of this, which are respectively accumulated in Alzheimer’s disease and Parkinson’s disease [146,147]. Moreover, the most suitable molecular target in curing/controlling the neurodegenerative diseases are located in brain parenchyma, being less accessible to the drugs inserted in the bloodstream (the most common way to administer drugs) due to highly selective permeability of BBB. Consequently, in such therapeutic procedures, the protection of brain parenchyma provided by endothelial cells of the BBB is not always desirable. For example, this barrier stops the entry of the necessary drugs in the treatment of neurodegenerative disorders (e.g., Parkinson’s, Alzheimer’s), which in turn were caused by functional alterations of the BBB [97,98]. To overcome this issue, a controlled increase in BBB permeability allowing a transient drug influx is a must for any drug-candidate targeting a molecular structure located in brain parenchyma. Therefore, drugs for neurodegenerative disorders must have a mechanism acting at the level of the BBB, which is why drug screening studies are based on the BBB study model, and for the study using microdevices, BBB-on-a-chip systems are used. 

The versatility of microfluidic chips to compartmentalize the biological model (NVU in this case) provides an optimal tool to design in vitro assays for NVU targeting drugs screening. The logical steps in analyzing the efficiency of such drugs can be easier run if each step is tested on an appropriate chip and only the best candidates, fulfilling the criteria of the step, are going to the next step.A series of microfluidic chips could provide a miniaturized low-cost platform for this drug screening flux.

The importance of pharmacological screening studies is given by the credibility and correctness of the data obtained in vitro compared to the in vivo processes [148]. The versatile potential of microdevices in the case of permeabilization and the BBB study has been demonstrated by several studies, which focused on mimicking neuroinflammation [97], the blood–tumor barrier study, with the possibility of developing strategies to allow testing of drugs for the purpose of treating this type of disease [149], mimicking the NVU and the cellular interactions that take place, in the presence of the drug substances [96], directly comparing the contact between vascular perfusable network and astrocytes [150].

**Figure 4:**
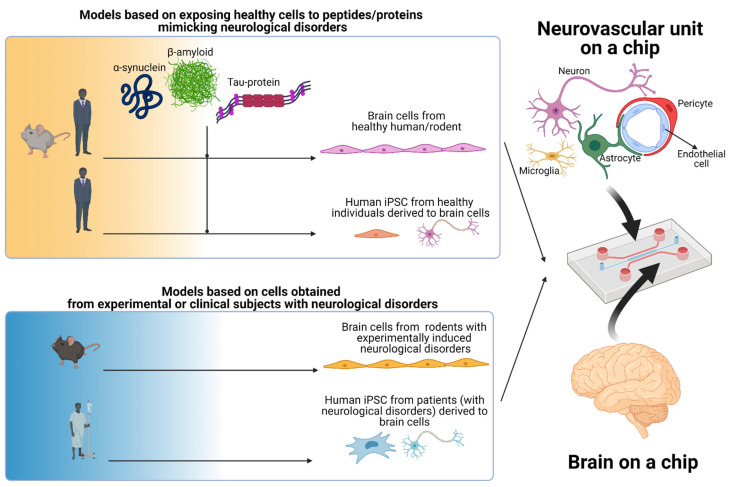
Strategies to mimic neurodegenerative disorders based on the NVU-on-a-chip/brain-on-a-chip technology. The main models developed to mimic neurodegenerative disorders on a chip are (i) testing human/rodent cells from healthy subjects exposed to peptides/proteins (e.g., beta-amyloid, alpha-synuclein, tau-protein), (ii) testing human/rodents cells obtained from experimental or clinical subjects with neurological disorders (generated with BioRender.com [91]).

Several strategies have evolved to mimic neurological disorders on a chip (Figure 4). The simplest in vitro model on a chip is to expose brain cells (e.g., cell lines) or neurospheroids of human or rodent origin to peptides/proteins, such as α-synuclein, β-amyloid, tau-protein etc., in order to obtain similar conditions at the BBB/NVU level that have elevated levels in brain parenchyma, cerebral spinal fluid, or cerebral blood flow associated with specific neurological pathologies [42,151,152]. In order to preserve the biochemical and morphological properties of brain cells, a good alternative is to cultivate on a chip primary cultures obtained from rodents or larger species (e.g., bovine, porcine, and nonhuman primate) [153]. These primary cultures can either be in vitro exposed to specific peptides/proteins that mimic the pathologies or can be obtained from animals in which experimental models have been developed/induced for specific neurological diseases. Alternatively, human-induced pluripotent stem cells (iPSC) derived from healthy subjects or from patients with neurological disorders can be used in BBB/NVU-on-a-chip in vitro models for neuropathologies.

Few studies reported the implementation of strategies based on LOC devices in NVU-targeted drug screening. In detail, the BBB permeability was tested for seven drugs: gabapentin, traxoprodil (mesylate), sertraline (hydrochloride), varenicline (tartrate), ethosuximide, sunitinib (malate), and c-secretase inhibitor; the results were compared with data obtained in animal studies, concluding the correctness of the data obtained using microfluidic platforms without the need to sacrifice animals [100]. Additionally, an organ-on-chip model of the human NVUwas used to mimic the effect of intravascular administration of the psychoactive drug methamphetamine by transiently opening the BBB [18]. 

## 6. LOC Microdevices in Translational Medicine with Impact in Neurological Disorders

Translational medicine represents the accumulation of several types of representative research activities in biomedical sciences, whose purpose is to optimize patient outcomes, screening and therapy of disease, disease prevention, and improving medical services by the rapid integration of new research results. The goal is to improve the working procedures and to develop new procedures for disease investigation [154], with a major impact on the quality of human life [155]. Through integrating the concept of personalized medicine in the larger field of translational medicine, the cutting-edge tool of biomedical research is figured out. 

There are great expectations regarding the use of LOCs and OOCs in biomedical applications, especially in translational medicine [156]. One of the potential advantages of LOC technology in the case of neuropathologies is the possibility of using this device in personalized medicine, thus being able to evaluate the effectiveness of a drug without following the treatment by the patient, in view of establishing the most suitable drug and its optimal concentration [110].

The key step in the translational process is from in vivo preclinical approaches, which are made usually on animal models, to early human clinical trials. In order to advance in this step, to extrapolate the research results obtained on animal models to human subjects, several issues should be considered, including the accuracy, reproducibility, accessibility, and expertise of the personnel dealing with this type of studies. The feedback is assured by indicators used to verify the results, such as biomarkers. Problems arise in situations where tests cannot be performed in parallel, because ethical rules can intervene. These types of tests are useful both in current translational medicine and in the development of devices useful for this purpose [157]. In this context, LOC microdevices are a good alternative to animal models. 

However, there are a number of limitations that, if overcome, will make LOC devices reliable tools in personalized medicine applications. Limitations that need to be overcome include aspect ratio, in the case of glass use, or geometry limitations in the case of polymer chips [158]. Other limitations include the difficulty to reproduce on a chip the complexity of the tissue architecture (e.g., brain tissue), the variability between protocols of human stem cell differentiation to target cells, the absence of immune cells that normally are activated in neuroinflammatory processes commonly associated with neurological diseases, etc. [73]. An important aspect that should be taken into account when projecting a BBB/NVU model based on an LOC platform is the difference in receptors/ion channels expression between primary cell cultures and cell lines with similar specie origin [159] or between species (i.e., human vs. rodent) [160]. Additionally, limitations can be due to the variability of the chip design depending on the purpose of the experiment, type of samples, throughput, or the experimental timeline [161]. In this regard, a detailed bioengineering approach of the BBB that considers the biomechanical and biochemical signaling in the neurovascular system [162] is an essential step to overcome the above-mentioned limitations. 

The results obtained based on NVU-on-a-chip devices are still difficult to be extrapolated in personalized medicine because of the way the brain receives, metabolizes, and is affected by drugs, neurotrophic agents, diseases, and pathogens, which involve BBB permeabilization [115].

A promising approach to obtain human brain-on-a-chip models that can be efficient tools in personalized medicine is to use human iPSC derived from patients with neurological disorders (Figure 4). Pioneering studies of human iPSC derived from healthy patients on a chip have been already done [69]. Moreover, there are several studies using human iPSC derived from patients with neurological disorders, such as Parkinson’s disease [163], Fragile X syndrome [164], schizophrenia [165], Dravet syndrome [166,167], Huntington’s disease [168], severe psychomotor retardation associated with the monocarboxylate transporter 8 [169], Alzheimer’s disease [170], Rett syndrome [171,172], Wilson’s disease [173], Timothy syndrome [174,175], etc., to unravel the mechanisms of disease. The next step will be to use autologous iPSCderived from patients onachip in personalized medicine. 

Microfluidic devices are considered in translational medicine studies by institutions such as the U.S. Food and Drug Administration (FDA) [176], which supports the initiative to develop such chips for clinical trials to help personalized therapies. A prerequisite of any new drug development is to overcome several important drug safety tests, which mandatory include its interaction with BBB (overcoming or not the barrier) [177] and its proarrhythmogenic risk [178,179]. Other safety tests, such as renal or liver toxicity, may also be considered. In this context, multi-organ-on-a-chip platforms could represent the best solution to integrate all drug safety tests in a single assay. 

To summarize, lab-on-a-chip/organ-on-a-chip technology has the potential to become an everyday tool to be used in current clinical practice for drug screening.

## 7. Conclusions

The development of microfluidic lab-on-a-chip and organ-on-chip systems has contributed to gain new insights in different biological fields, particularly in the neuroscience domain. Further advancements are expected to downsize architectures to nanoscale and provide relevant 3D in vivo configurations for specific applications, including the investigation of biological sub-cellular aspects and chemical analysis with improved molecular detection limits. 

Although brain-on-a-chip models brought new scientific insights and in some respects have a translational potential when human stem derived cells are used, these models are rather limited, as neurospheroids represent an oversimplified representation of the human brain. Therefore, new and more sophisticated bioengineered configurations of human brain-on-a-chip platforms will enable a step forward in mimicking neurological diseases based on iPSC derived from patients affected by a certain neuropathology and, thus, enabling in the near future patient-oriented drug screening and biomarker detection. If current technical limitations will be overcome, then it will be created the premises of using microfluidic LOC platforms in personalized medicine.

There are yet important issues to be resolved for biomedical applications, in particular the lack of standardization as it concerns material selection, process development, and tool design to reach the desired goal [180]. The material properties should be well correlated either with fabrication methods or biological purposes. Then, manufacturing technology should offer flexibility in design and prototyping for a robust device development. It is concluded that LOCs and OOCs may already challenge preclinical studies, yet academic researchers, the pharmaceutical industry, and regulatory authorities should collaborate closer in order to fulfill its potential [144].In conclusion, if all the stakeholders will contribute, the LOC/OOC technology has the potential to be become an everyday tool to be used in current clinical practice for drug screening.

## Figures and Tables

**Table 1:** Properties of materials and processing technologies typically used for the fabrication of microfluidic devices (adapted from [11]).

Material/Property	Silicon/Glass	Elastomers	Thermosets	Thermoplastics	Hydrogel	Paper
optical transparency	no/high	high	high	medium to high	low to medium	low
hydrophobicity	hydrophilic	hydrophobic	hydrophobic	hydrophobic	hydrophilic	amphiphilic
thermostability	very high	medium	high	medium to high	low	medium
resistance to oxidizer	excellent	moderate	good	moderate to good	low	low
solvent compatibility	very high	low	high	medium to high	low	medium
permeability to oxygen (Barrer ^a^)	<0.01	≈500	0.03–1	0.05–5	>1	>1
surface charge	very stable	not stable	stable	stable	N/A	N/A
**common technique for microfabrication/features**	**photolithography, laser-assisted etching**	**casting**	**casting, photopolymerization**	**thermo-molding**	**casting, photopolymerization, 3D bioprinting**	**photolithography, printing**
smallest channel dimension	<100 nm	<1 μm	<100 nm	≈100 nm	≈10 μm	≈200 μm
channel profile	limited 3D/3D	3D	arbitrary 3D	3D	3D	2D
multilayer channels	hard/easy	easy	easy	easy	Medium	easy
throughput	medium to high	high	high	high	low to medium	high

^a^ Barrer = 10^−10^ [cm^3^ O_2_(STD)] [12] cm cm^−2^ s^−1^ cmHg^−1^.

**Table 2:** Organ-on-a-chip applications.

Organ/Tissue Type	Chip Material	Membrane Material	Application	Reference
Alveolus-on-a-chip	PDMS	PDMS	Interface alveolar epithelium/endothelium for the study of inflammation-induced thrombosis	[60]
Bone marrow-on-a-chip	PDMS	PDMS	Analysis of the cellular response to drugs and radiation	[61]
Gut-on-a-chip	PDMS	Polyester	Development of a platform for drug screening and substance toxicity testing	[62]
Heart-on-a-chip	PDMS	No membrane	Testing the inotropic effect of isoproterenol on cardiac contractility	[63]
	PMMA and PDMS	No membrane	Evaluation of cardiovascular toxicity of some pharmaceutical products	[64]
Intestine–liver–brain–kidney-on-a-chip	PDMS	PDMS	Production and testing of an autologous iPSC derived four-organ-on-a-chip in long-term cocultivation conditions (i.e., 14 days)	[69]
Kidney-on-a-chip	PDMS	Polyester	Analysis in conditions close to the physiological ones of renal tubule cells	[70]
Lung-on-a-chip	PDMS	PDMS	Mimicking and analyzing the long alveolar barrier	[65]
Lung–liver–heart-on-a-chip	PMMA and PDMS	Polyester	Assessment of the importance of interactions between organs in response to drugs	[68]
Pancreas-on-a-chip	PDMS	Polyester	Investigating the role of CFTR (Cystic Fibrosis Transmembrane Conductance Regulator) in insulin production	[66]
Skin-on-a-chip	PDMS	Polyester	Mimicking edema and inflammation of the skin and testing dexamethasone effects	[67]

**Table 3:** PDMS microfluidicbrain-on-a-chip platforms.

Organ/Tissue Type	Type of Cells	Application	Reference
Brain organoid-on-a-chip	3D brain organoids derived from human-induced pluripotent stem cells (hiPSCs)	Modeling the neurodevelopmental disorders under environmental exposure (e.g., nicotine)	[77]
3D brain-on-a-chip	Neurospheroids obtained from prenatal E16 rat cortical neurons	In vitro brain model for neurodegenerative disease (e.g., Alzheimers’ disease) and high-throughput drug screening	[42]
Brain-on-a-chip	Neurospheroids obtained from human neural progenitor and human iPSC-derived neural progenitor cells	Investigating the development of Alzheimer’s disease and testing drugs against this neuropathology	[78]
Neurospheroid network-on-a-chip	Neurospheroids obtained from primary culture obtained from the cerebral cortex of Wistar rats	Studying neural transplantation therapy for treating severe degenerative brain disease	[79]
3D brain-on-a-chip	Neurospheroids obtained from prenatal rat (E18) cortical neurons	Modulation of cell–ECM interactions at the neuronal level by analyzing neurospheroids and their study in pathological conditions	[80]

**Table 4:** BBB-on-a-chip and NVU-on-a-chip applications.

Model	Chip Material	Membrane Material	Culture Type	Cells	Application	Reference
BBB	PDMS and glass	Polycarbonate	Co-culture	Endothelial cells (b.End3) and astrocytes (C8D1A)	BBB permeability	[100]
BBB	PDMS	Polyethylene terephthalate	Co-culture	Endothelial cells (BMEC from hiPCS) and astrocytes (from IMR90-4 iPSCs)	BBB permeability due to TNF-α in liver failure/melanoma	[101]
BBB	OrganoPlate	No membrane	Tri-culture	Endothelial cells (TY10), astrocytes (hAst) and pericytes (hBPCT)	BBB permeability for different types of molecules (antibodies)	[98]
BBB	Objet Vero Clear, silicone, and PDMS	Polycarbonate	Co-culture	Endothelial cells (BMEC from iPSC) and astrocytes (Rat primary culture)	BBB permeability for drugs	[102]
BBB	PDMS	Polycarbonate	Co-culture	Primary mouse brain microvascular endothelial cells and primary mouse astrocytes	Cellular interactions in the BBB under physiological or shear stress conditions	[103]
BBB	PDMS	Polyester and polytetrafluoroethylene	Co-culture	Endothelial cells (b.End3) and astrocytes (C8D1A)	Analysis of cell cultures on porous membranes	[104]
BBB	PMMA	Polyester	Monoculture	Endothelial cells (b.End3)	Transport of nanoparticles across the BBB	[105]
BBB	PDMS and polyvinylidene fluoride (PVDF)	Polyvinylidene fluoride (PVDF)	Co-culture	Human cerebral microvascular endothelial cells (hCMEC/D3) and normal human astrocytes	Reproducible platform for the BBB study under static or continuous flow conditions	[106]
BBB	PDMS	Polycarbonate	Tri-culture	Human cerebral microvascular endothelial cells (HBMEC), pericytes, and astrocytes	BBB model for the investigation of neuroinflammation	[107]
BBB	PDMS	No membrane	Multi-Culture	Endothelial cells (HBMEC and HUVEC), pericytes (HhPC-PL), astrocytes (NHA), and primary normal human lung fibroblasts (LF)	In vitro reproduction of angiogenesis in the central nervous system	[108]
BBB	PDMS, PMMA, and PC	N/A	Co-culture	Endothelial cells (HUVEC) and human astrocytes	Testing the biocompatibility of the APTES-coated PDMS surface, on which different types of coating were applied	[109]
NVU	PDMS	No membrane	Tri-culture	Human iPSC-derived blood–brain barrier cells Human primary astrocytes Human primary pericytes	Complex platform for the study of neurological diseases	[110]
NVU	PDMS	PDMS	Co-culture (×2)	Human teratocarcinoma NTERA-2 cl. D1 (hNT2) cells and human endothelial cells (hBMEC) Human teratocarcinoma NTERA-2 cl. D1 (hNT2) cells and Human fetal neural progenitor cells (hNPCs)	Differentiation of cells on the chip and analysis of the importance of cell interactions in neurodevelopment	[111]
NVU	PDMS	No membrane	Multi-Culture	Endothelial cells (HUVEC and hCMEC/D3), neurons (primary culture), and astrocytes (primary culture)	Neurovascular unit development	[17]
NVU	PDMS andpolycarbonate	Polyethylene terephthalate andpolycarbonate	Multi-Culture	Human hippocampal neural stem cells HIP-009 cells, cortical human brain microvascular endothelial cells (hBMVECs), human astrocytes, and human brain pericytes of cortical origin	Effect of intravascular administration of methamphetamine	[18]

**Table 5:** Comparison between the BBB-on-a-chip and BBB in the transwell system.

Type of Analysis	Comparison	Type of Cells	References
TEER ZO1 immunostaining	Slightly higher resistance values upon 7 days in culture for BBB-on-a-chip compared to the transwell system Similar ZO1 immunostaining	human endothelial cells hCMEC/D3	[97]
TEER ZO1 immunostaining	Astrocyte conditioned medium improves the resistance values of BBB-on-a-chip BBB-on-a-chip has higher resistance values than the transwell model	Rat brain endothelial cells (RBEC) isolated from neonatal rats neonatal rat astrocytes	[113]
TEER	μBBB had significantly higher (10-fold) resistance values than the transwell model for co-cultures	b.End3 endothelial cells, with and without co-cultured C8-D1A astrocytes	[100]
Barrier permeability and cytokine release profile	Similar permeability of the human 3D BBB-on-a-chip compared to the non-human cells BBB models or to the inflammatory stimulated models (depending on the presence of astrocytes or pericytes) Significantly higher permeability of the human 3D BBB-on-a-chip compared to co-cultures in static transwell plates	Co-culture of human brain microvascular endothelial cells, human brain pericytes, human astrocytes (from cortex)	[95]
P-glycoprotein (P-gp) permeability	BBB-on-a-chip model, but not the transwell model, enable the study of P-gp efflux pump permeability and its pharmacological blockade (e.g., verapamil)	Human iPS cell line IMR90-4	[23]

## Data Availability

Not applicable.
